# Surface Morphology, Compressive Behavior, and Energy Absorption of Graded Triply Periodic Minimal Surface 316L Steel Cellular Structures Fabricated by Laser Powder Bed Fusion

**DOI:** 10.3390/ma15238294

**Published:** 2022-11-22

**Authors:** Bharath Bhushan Ravichander, Shweta Hanmant Jagdale, Golden Kumar

**Affiliations:** Mechanical Engineering, University of Texas at Dallas, Richardson, TX 75080, USA

**Keywords:** laser powder bed fusion, 316L steel, porous structure, triply periodic minimal surface, deformation behavior, energy absorption, surface morphology

## Abstract

Laser powder bed fusion (LPBF) is an emerging technique for the fabrication of triply periodic minimal surface (TPMS) structures in metals. In this work, different TPMS structures such as Diamond, Gyroid, Primitive, Neovius, and Fisher–Koch S with graded relative densities are fabricated from 316L steel using LPBF. The graded TPMS samples are subjected to sandblasting to improve the surface finish before mechanical testing. Quasi-static compression tests are performed to study the deformation behavior and energy absorption capacity of TPMS structures. The results reveal superior stiffness and energy absorption capabilities for the graded TPMS samples compared to the uniform TPMS structures. The Fisher–Koch S and Primitive samples show higher strength whereas the Fisher–Koch S and Neovius samples exhibit higher elastic modulus. The Neovius type structure shows the highest energy absorption up to 50% strain among all the TPMS structures. The Gibson–Ashby coefficients are calculated for the TPMS structures, and it is found that the C_2_ values are in the range suggested by Gibson and Ashby while C_1_ values differ from the proposed range.

## 1. Introduction

The advances in computer-aided design (CAD) and additive manufacturing (AM), have led to the realization of complex geometries in metallic materials [1,2]. The new design concepts, such as topology optimization, can enable the reduction in weight without compromising the performance of a metal part. The combination of AM techniques and topology optimization has resulted in fabrication of light weight triply periodic minimal surface (TPMS) structures with high energy absorption efficiency, better vibration reduction, and good biocompatibility [3,4]. The TPMS structures are used as shock absorbers in spacecrafts and high-speed trains due to their high energy absorption capabilities [5,6]. LPBF is one of the most widely used metal AM techniques to fabricate complex metal TPMS structures with high accuracy [7,8,9,10,11]. The LPBF is a layer-by-layer manufacturing process in which the laser beam selectively melts and joins the metal powder layers until the desired part is fabricated [11,12,13].

The TPMS structures are classified as bending- or stretching-dominated. The bending-dominated TPMS structures like Tetrakaidecahedron and BCC display longer stress plateau regions, whereas the stretching-dominated TPMS structures like octet-truss soften after yielding [14,15]. The desired yield strength and Young’s modulus can be obtained by optimizing the size and the geometry of the unit cells in metal TPMS structures. The periodic unit cells in TPMS structures are typically defined by using mathematical equations. These equations allow controlling the pore size, the zero-mean curvature, and the surface area in TPMS unit cells. Diamond, Gyroid, and Schwartz are the commonly used unit cell types in metal TPMS structures. Numerous studies have been conducted to understand the correlation between the unit cell characteristics and the mechanical properties of metal TPMS structures [16,17,18]. Gibson et al. established a model to determine the effect of relative density on the mechanical properties of the TPMS structures [14]. Al-Saedi et al. conducted a similar study and determined the Gibson–Ashby co-efficients for functionally graded F2BCC lattice structure with the help of experiments and calculations [19]. The TPMS structures with smooth and continuous curves are known for their high-strength and energy absorption capabilities [20,21]. Likewise, Al-Ketan et al. concluded that the sheet-based TPMS structures tend to show a higher energy absorption and better load bearing capacity compared to solid TPMS structures [22].

316L steel has been extensively studied using LPBF because of its widespread use in structural and functional applications [23]. Bonatti et al. compared the shell-based TPMS structures with that of truss lattices in 316L steel and found that the TPMS structures showcased superior mechanical properties compared to the truss lattices with the same density [24]. Yang et al. compared the compressive behavior of SLM-manufactured 316L steel uniform and graded Gyroid lattices. It was established that the graded lattices had improved mechanical properties compared to the uniform Gyroid lattices [25]. In a similar study conducted by Yang et al., the effect of sandblasting on the compressive and fatigue behavior of uniform 316L steel Gyroid lattice, it was found that the overall mechanical behavior of the Gyroid sample improved after sandblasting [26]. The surface morphology, mechanical response, and microstructure of uniform TPMS structures have been extensively studied [27,28,29]. 

The relationship between mechanical properties and grading approaches for functionally graded TPMS samples have been studied [25,30,31,32,33]. The mechanical properties of functionally graded Diamond sample was investigated by Han et al. [33]. They found that the functionally graded nature of the diamond sample led to layer-by-layer failure mechanism. This is different from the uniform Diamond structure where the sample failed due to the formation of diagonal shear bands. In a study conducted by Liu et al. [31], the effect of changing cell type, cell size, and relative density was investigated. Acceleration of biodegradation of functionally graded TPMS structures were reported by Li et al. [32]. They found that the topology of TPMS lattices improved the degradation of metals. However, the functionally graded TPMS structures are underexplored especially in structures like Fisher–Koch S and Neovius compared to Gyroid, Diamond, and Primitive structures. 

The aim of this study is to analyze the surface morphology, the mechanical properties, and the energy absorption of functionally graded TPMS structures such as Gyroid, Diamond, Primitive, Neovius and Fisher–Koch S fabricated from 316L steel using LPBF. The relative density of the structures was designed to vary from 30% to 70%. The samples were sandblasted to improve the surface finish and compressive tests were performed to measure the mechanical response and energy absorption capability. Further, Gibson–Ashby model was implemented to determine the pre-factor coefficients C_1_ and C_2_.

## 2. Fabrication and Experimental Procedure

### 2.1. Design of TPMS Structures

**Figure 1:**
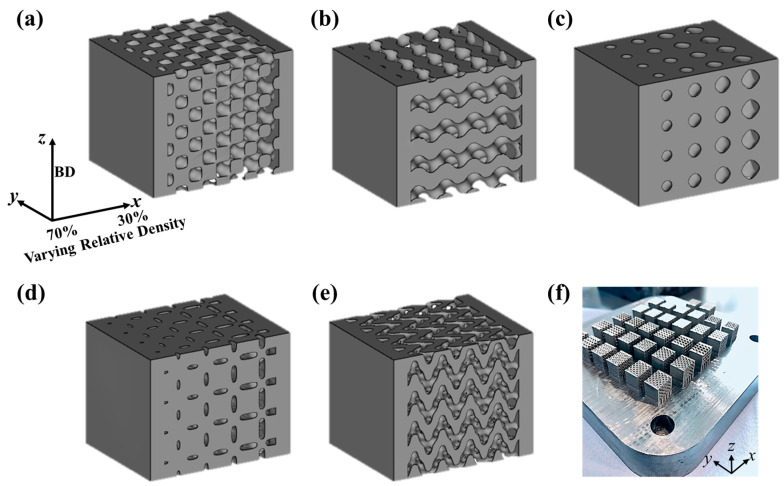
Designed TPMS structures with relative density varying from 30% to 70% and build direction (BD) indicated by the arrow and types: (**a**) Diamond; (**b**) Gyroid; (**c**) Primitive; (**d**) Neovius; (**e**) Fisher–Koch; (**f**) As-built samples on the build plate.

As shown in Figure 1, five TPMS structures (i.e., Diamond, Gyroid, Primitive, Neovius, and Fisher–Koch S) were designed by using an open source TPMS generator MS Lattice, [34]. The size of the samples was maintained at 8 mm × 8 mm × 8 mm with each unit cell being 2 mm × 2 mm × 2 mm. The relative density of the samples varied from 30% to 70% along the x-direction as shown in Figure 1. The TPMS designs were converted into 3D stereo-lithography (STL) files, which were imported into Solidworks. Two end plates, each with the thickness of 1 mm, were added at the top and the bottom of the structures to support the compressive loading. The files were then imported to Materialise Magics (© Copyright Materialise 2021, Leuven, Belgium) to assign the supports and the corresponding process parameters for 3D printing. Equations (1)–(5) represent the nodal approximations of the TPMS structures, and their porosity is governed by the constant *K* as defined by Al-Ketan et al. [34]:

Diamond: *f* (*x*, *y*, *z*) = *sin*(*x*) × *sin*(*y*) × *sin*(*z*) + *sin*(*x*) × *cos*(*y*) × *cos*(*z*) + *cos*(*x*) × *sin*(*y*) × *cos*(*z*) + *cos*(*x*) × *cos*(*y*) × *sin*(*z*) − *K* (1)

Gyroid: *f* (*x*, *y*, *z*) *= sin*(*x*) × *cos*(*y*) + *sin*(*y*) × *cos*(*z*) + *sin*(*z*) × *cos*(*x*) − *K* (2)

Primitive: *f* (*x*, *y*, *z*) *= cos*(*x*) + *cos*(*y*) + *cos*(*z*) − *K* (3)

Neovius: *f* (*x*, *y*, *z*) *=* 3 × (*cos*(*x*) + *cos*(*y*) + *cos*(*z*)) + 4 × *cos*(*x*) × *cos*(*y*) × *cos*(*z*) *− K* (4)

Fisher–Koch S: *f* (*x*, *y*, *z*) *= cos*(2*x*) × *sin*(*y*) × *cos*(*z*) + *cos*(2*y*) × *sin*(*z*) × *cos*(*x*) + *cos*(2*z*) × *sin*(*x*) × *cos*(*y*) *− K* (5)

### 2.2. Powder Preparation and Fabrication

**Figure 2:**
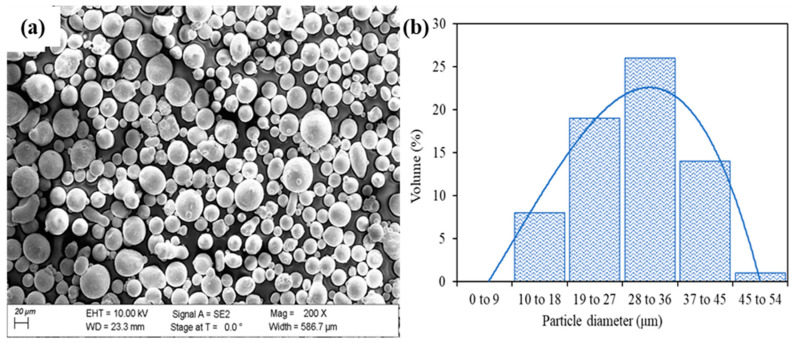
(**a**) SEM micrograph of powder; (**b**) particle size distribution of commercial SLM solutions 316L powder.

The TPMS structures shown in Figure 1 were fabricated from 316L steel using LPBF printer SLM 125 HL (SLM Solutions Group AG, Lübeck, Germany). The printer is equipped with a 400 W Ytterbium fiber laser and has a building volume of 125 mm × 125 mm × 125 mm. A total of three samples for each TPMS design were fabricated as shown in Figure 1f. The scanning electron microscopy (SEM) micrograph of the gas-atomized 316L steel powder obtained from SLM solutions AG is shown in Figure 2a. Image J [35] software was used to determine the particle size of the fresh powder and a histogram of the particle size distribution was obtained as seen in Figure 2b. The average powder particle size was found to be 30 µm. The composition of the fresh 316L steel powder is listed in Table 1.

The laser power (LP = 200 W), scan speed (SS = 800 mm/s), hatch spacing (HS = 120 μm), layer thickness (LT = 30 μm), and the stripes scan strategy were used for the fabrication of 316L steel TPMS samples. The above-mentioned process parameters were suggested by the manufacturer SLM solutions Group AG. The energy density (*E_v_*) used during the fabrication process is 69.45 J/mm^3^, which is calculated as [10,13,36]:

(6) $$E_{v}=\frac{LP}{SS\times HS\times LT}$$

### 2.3. Experimental Procedure

A wire electrical discharge machine was used to remove the as-built TPMS samples from the steel substrate. The samples were ultrasonically cleaned in iso-propyl alcohol. Subsequently, the samples were sandblasted using a Shop Fox M1114 benchtop Sandblaster and 100 grit aluminum oxide sand with a pressure of 60 psi for 30 s on each side to remove the unmelted powder particles uniformly from all sides. Surface morphology was analyzed using a Keyence VHX-970FN digital microscope.

Density measurements were performed using the Archimedes method as described by Ma et al. [27]. A Veritas Precision Balance with a sensitivity of 0.0001 g was used for the density measurements. The room temperature quasi-static compression tests were conducted using an Instron 5969 universal testing machine equipped with a 50 kN load cell. A constant strain rate of 10^−3^ sec^−1^ was applied along the x-direction (30% dense side) for a maximum displacement maintained at 50% for each sample. Using the load vs. extension data from the compression test, the stress vs. strain curves were obtained. The stress values were calculated by dividing the force by the cross-sectional area of the sample and the strain was determined by dividing the displacement by the sample height. A total of 3 samples were tested for each TPMS design and the average values for yield strength and Young’s modulus are reported.

## 3. Results and Discussion

### 3.1. Surface Morphology and Ensity Analysis

**Figure 3:**
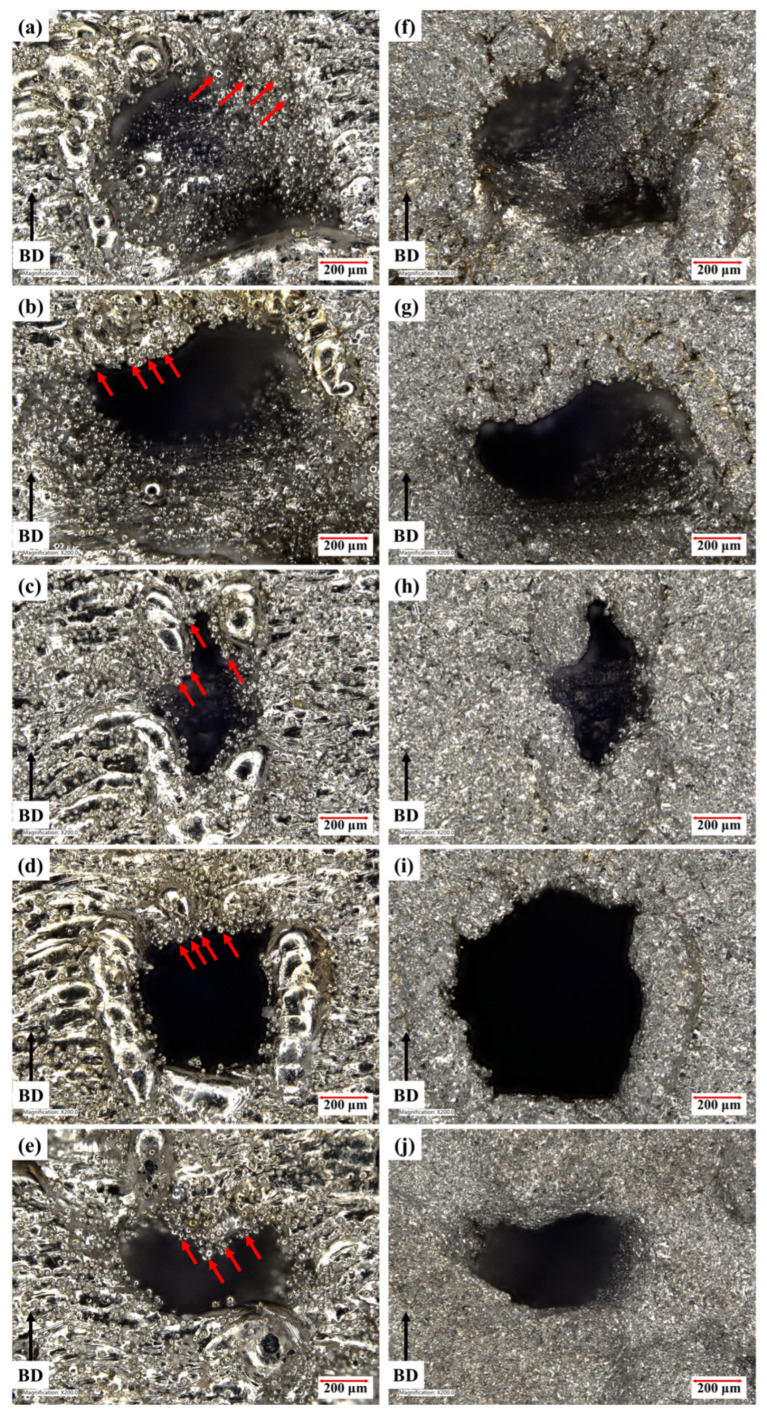
Optical micrographs along the build direction showing the unmelted powder particles highlighted with arrows attached to the surface for the as-built samples (**a**) Diamond, (**b**) Gyroid, (**c**) Neovius, (**d**) Primitive, and (**e**) Fisher–Koch S and the corresponding surfaces after sandblasting for (**f**) Diamond, (**g**) Gyroid, (**h**) Neovius, (**i**) Primitive, and (**j**) Fisher–Koch S samples.

The surface finish and the density of the TPMS samples significantly affect their mechanical properties [37]. Thus, it is important to ensure that the samples are fabricated without any large defects. The optical micrographs of the as-built TPMS samples are shown in Figure 3a–e. In general, no macroscale defects were observed in all the samples. However, rough surfaces were found along the struts due to the presence of unmelted powder particles on the side and the overhang surfaces. The unmelted powder particles form during the laser scanning which leads to the partial melting of adjacent powder particles on the side and the overhang surfaces along the designed path (highlighted with red arrows) as shown in Figure 3a–e. 

**Figure 4:**
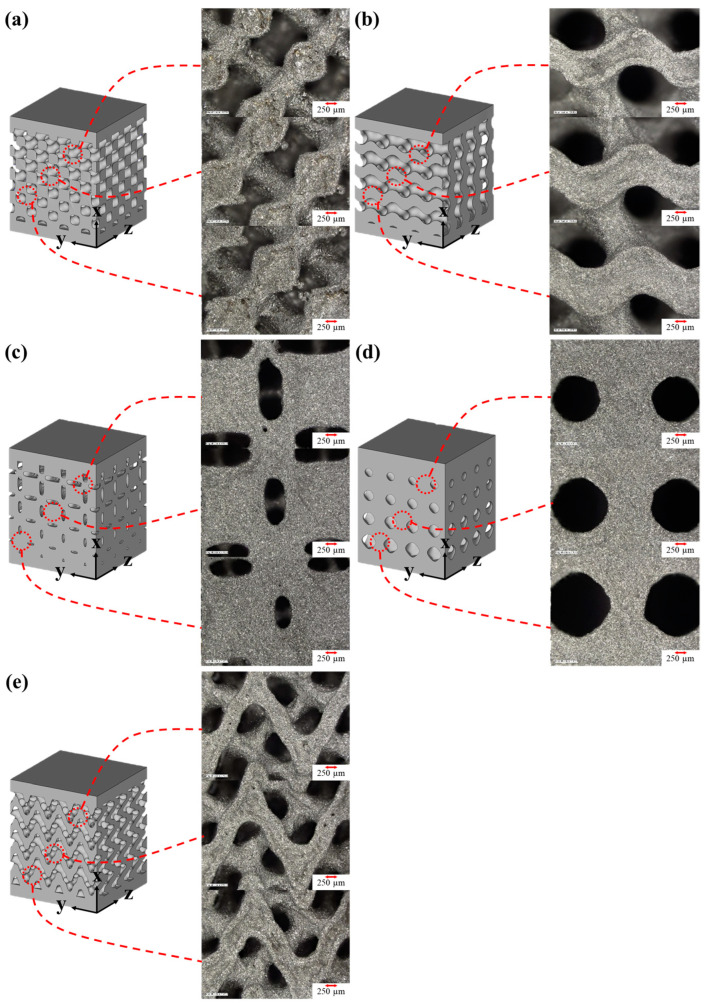
Surface morphologies of LPBF (**a**) Diamond, (**b**) Gyroid, (**c**) Neovius, (**d**) Primitive, and (**e**) Fisher–Koch S type TPMS samples after sandblasting.

The samples were sandblasted to remove the unmelted powder particles and improve the surface finish of the TPMS samples as the presence of unmelted powder particles on LPBF samples can be detrimental to the mechanical behavior [26]. From Figure 3f–j, we can observe that the sandblasting effectively removed the unmelted powder particles. The surfaces of sandblasted TPMS samples at different regions are shown in Figure 4. The optical micrographs confirm that there are no macroscale defects such as cracks, pores, and deformation, thus indicating the good manufacturability of the TPMS structures using LPBF technique. One can also notice a gradual change in the dimensions of the struts as the part density changes from 70% porous at the top to 30% porous at the bottom.

The TPMS samples were cleaned In an ultrasonic bath after sandblasting and a precision balance was used to measure the mass of the samples in air and water. The part densities of the samples were calculated which are presented in Table 2. The achievement of near fully dense samples is usually expected in solid parts fabricated by the LPBF process due to layer-by-layer melting. Due to the functionally graded nature of the TPMS specimens and the small unit cell size of 2 mm, it was noticed that the part density values were lower, which is consistent with the literature [38]. The lower part density values can be due to shrinkage, lack of fusion between powder particles, and unmelted powder particles in the more porous (70% porous side) region consisting of larger overhang sections and thermal stresses as it was fabricated without support structures [16,39]. We observed that at more porous regions, there are some discontinuities in the struts of the samples leading to weak intersections as the features are closer to the threshold of the laser spot size. The melt pool size in LPBF technique is larger than the spot size of the laser. Thus, the scan contour tracks compensate for this by shifting inwards as reported by [40]. The contour tracks also partially melt the powder particles adjacent to the walls and therefore cause more powder particles to adhere to the surface. These process-related factors lead to marginal increase or decrease in the thickness of the struts which further justifies the small deviations in the part density of the samples as also noticed by Al-Ketan et al. [41].

### 3.2. Quasi-Static Compression Analysis

**Figure 5:**
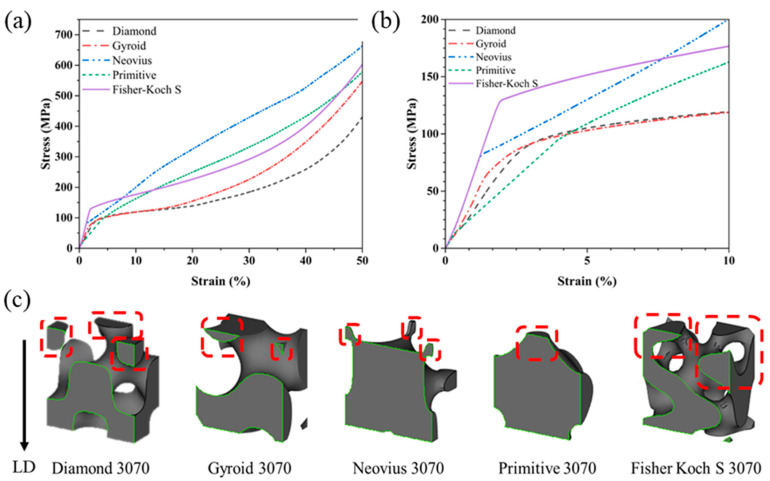
(**a**) Stress–strain curves of the different functionally graded lattice specimens from 0 to 50% strain. (**b**) Stress–strain curve of the fabricated samples with 0 to 10% strain. (**c**) Section views of the unit cells with loading direction (LD).

**Figure 6:**
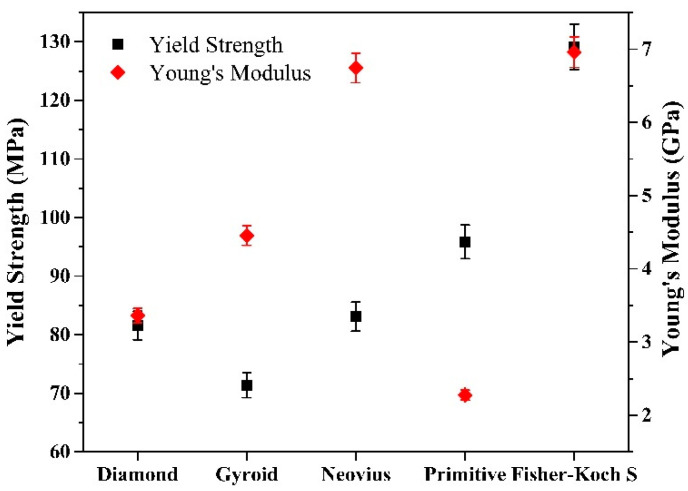
Young’s Modulus and yield strength of the different functionally graded lattice samples.

The compressive stress–strain curves of the TPMS samples are shown in Figure 5a (up to 50% strain) and Figure 5b (up to 10% strain). The tests were performed with the loading direction perpendicular to the build direction (see Figure 1). The Young’s modulus was obtained by calculating the slope of the linear elastic region and the compressive yield strength was determined by using 0.2% offset approach [39]. The stress–strain curves indicate that after initial elastic region, the structures deform plastically and continue to absorb energy and it is followed by the densification stage, where the samples behave like a solid material as there is a large self-contact area. A similar deformation behavior was observed by Li et al. for 316L steel Gyroid structures [16]. Figure 6 compares the Young’s modulus and yield strength values for the 5 TPMS structures. The Fisher–Koch S specimen shows the highest Young’s modulus value of 6.96 GPa followed by the Neovius (6.74 GPa) and Gyroid (4.46 GPa) samples. The Diamond and the Primitive samples exhibit lower Young’s modulus of 3.36 GPa and 2.28 GPa, respectively. The Fisher–Koch S sample has the highest yield strength of 129 MPa. The Primitive sample yields at 95.9 MPa. The Diamond and Neovius type samples have similar yield strength values in the range of 81–83 MPa and the Gyroid sample exhibits the lowest yield strength of 71.3 MPa. 

The section views of the designed unit cells along with the loading direction are shown in Figure 5c. The stress–strain curve in Figure 5b indicated that the Gyroid sample has the lowest yield strength compared to the other samples and this can be attributed to the lack of structural continuity to the upward facing surface (highlighted in red). As the load is applied, the highlighted surface is not able to transfer the load to the layers below it and thus yields at a strain value of 1.7%. A similar trend can be observed for Diamond (2.6% strain) and the Neovius (1.4% strain) samples, where there is a lack of continuity between the struts and the main portion of the respective designs. The Primitive and the Fisher–Koch S samples have better structural continuity between the upward-facing surfaces and the rest of the main portion of the TPMS design. Thus, the Fisher–Koch S and Primitive TPMS designs have higher yield strength values as the load is transferred across the samples more uniformly compared to the other TPMS samples. The Young’s modulus of functionally graded materials with loading applied parallel to the grading direction can be predicted by applying the rule of mixtures such that [41]:

(7) $$\frac{1}{E_{graded}}=\frac{1}{m}\sum_{i=1}^{m}\frac{1}{E_{i}}$$

Here, the cross-sectional area is assumed to be constant and unchanged throughout the loading direction, and the thickness of each layer is L/m where L is the total length of the specimen and m is the number of layers. This has been discussed in detail by Maskery et al. [42].

**Figure 7:**
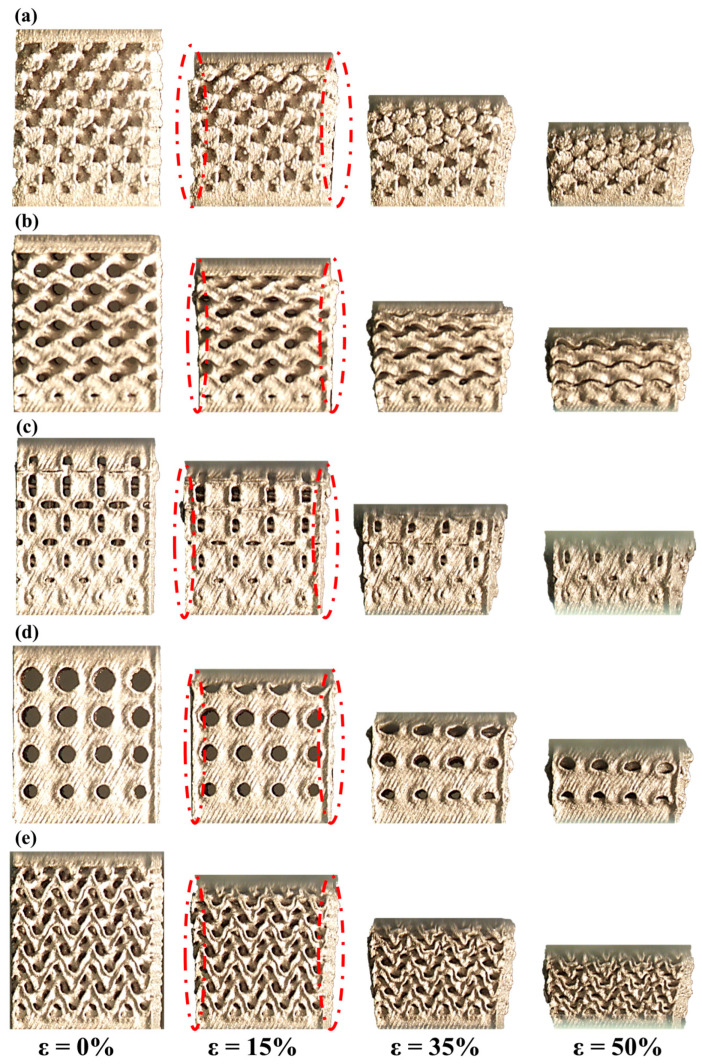
Images of (**a**) Diamond, (**b**) Gyroid, (**c**) Neovius, (**d**) Primitive, and (**e**) Fisher–Koch S TPMS structure recorded at different strains.

The deformation of samples at different strains (ε) was captured by recording the images as shown in Figure 7. During the first 15% strain, the samples expand laterally by forming a radius along the edge of the TPMS cubes as shown in Figure 7 (highlighted with dotted lines for the 15% strain images). The plastic deformation starts at about 1.1% and 1.2% strain for the Gyroid and Neovius samples, respectively. Whereas the plastic region for the Diamond and the Fisher–Koch S samples begins at 1.3% and 2% strains. The Primitive sample has the largest elastic deformation range up to 4% strain. Until 50% strain, no sudden reduction in stress is observed indicating there is no brittle fracture during compression. The samples did not deform with a diagonal shear, but rather deformed layer-by-layer as observed in Figure 7. Similar findings were reported by Yang et al. and Han et al. [25,33].

The Young’s modulus and yield strength for different relative densities of metallic foams can be predicted using the Gibson–Ashby model [14]. The formulae of the Gibson–Ashby model are as follows:

(8) $$\frac{E}{E_{o}}=C_{1}{\left(\frac{\rho }{\rho_{o}}\right)}^{2}$$

(9) $$\frac{\sigma }{\sigma_{o}}=C_{2}{\left(\frac{\rho }{\rho_{o}}\right)}^{1.5}$$

where *E*, *ρ*, and *σ* are the apparent compressive modulus, the density, and the compressive yield strength of open-cellular structures, respectively. *E_0_*, *ρ_0_*, and *σ_0_* are the respective values for the fully dense materials and *C*_1_ and *C*_2_ are Gibson–Ashby coefficients. Bulk compressive yield strength and elastic modulus of 316L steel alloy are taken to be 205 MPa and 193 GPa, respectively [2]. The *E*, *ρ*, and *σ* for the TPMS structures were determined from the experimental stress–strain measurements. The determined values for *C*_1_ and *C*_2_ for every sample are listed in Table 3. The obtained *C*_1_ and *C*_2_ values can be used in further studies for the TPMS structures with porosity levels varying from 30% to 70% without mechanical testing. The values for *C*_1_ and *C*_2_ depend on the material and the lattice type. From Table 3, we can observe that the *C*_1_ values are out of the range given by Gibson and Ashby. This can be attributed to the differences in the parameters of the LPBF process as also noted by Zhao et al. and Yan et al. [21,43]. It is well known that the process parameters of the LPBF process play a significant role in determining the properties of the as-built specimens as discussed extensively by Ravichander et al. [8,12]. Additionally, the range of values defined by Gibson and Ashby was established for the mechanical properties of metallic foams. The *C*_2_ values agree with the range given by Gibson and Ashby.

### 3.3. Energy Absorption

**Figure 8:**
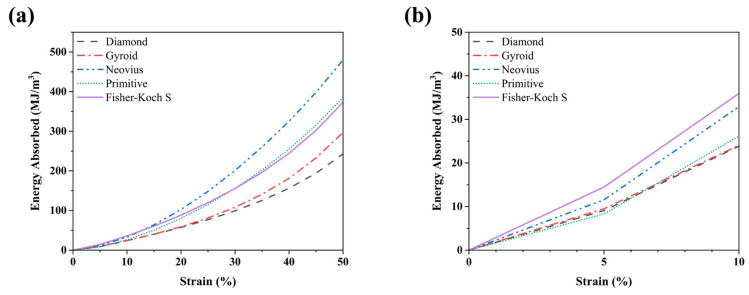
(**a**) Energy absorption–strain curves for the different functionally graded lattice specimens from 0 to 50% strain. (**b**) Energy absorption–strain curves of the fabricated samples with 0 to 10% strain.

The lattice structures are known for their low density and high energy absorption capabilities. Thus, they can be used in protective devices, implants, and in aerospace components. The cumulative energy absorption per unit volume (*W_v_*) is widely utilized to determine the energy absorption capability of the lattice structures. The energy absorption per unit volume up to 50% strain was calculated using the following equation:

(10) $$W_{v}=\overset{0.5}{\underset{0}{\int }}\sigma \left(\varepsilon \right)d\varepsilon$$

where *ε* is the strain, and *σ*(*ε*) is the stress related to ε during the compression test. According to the ISO13314 standard [44], compressive stress–strain curves were integrated for the energy absorption characterization. Origin was used to integrate the area under the stress–strain curves. The cumulative energy absorption–strain curves are shown in Figure 8a, and the total energy absorbed values are presented in Table 4. It can be observed from Figure 8a that the cumulative energy absorption increases steadily, which is then followed by an exponential increase due to the increase of density as well as the structural stiffness after the layers collapse. From Table 4, we can note that the Neovius (479.62 MJ/m^3^) sample absorbed the most energy followed by the Primitive (384.95 MJ/m^3^) and Fisher–Koch S (373.08 MJ/m^3^) samples. The Gyroid (296.67 MJ/m^3^) and the Diamond (242.90 MJ/m^3^) samples absorbed the second lowest and lowest energy per unit volume, respectively. From the sectional view of the Neovius design in Figure 4c, we can see that the highlighted portion of the struts collapse leading to the densification of the sample, and it is further supported from Figure 5a, where we can notice that the Neovius sample has a small plateau stress region and larger densification region compared to the other samples. A similar trend can be observed for the Fisher–Koch S and Primitive samples. On the contrary, the Diamond and the Gyroid TPMS samples have a larger plateau stress region and start densifying around the 15% strain mark. The energy absorption capabilities depend significantly on the TPMS structure and its deformation behavior than the density [4]. This can be observed in the initial energy absorption–strain curves presented in Figure 8b as the Fisher–Koch S sample absorbs more energy at the beginning and then the Neovius sample absorbs more energy from the 12.5% strain value. From Figure 8a, it can be observed that the *W_v_* values gradually increase for all TPMS samples during compression, and it is due to the layer-by-layer deformation behavior with improved load-bearing capabilities to absorb more energy during the compression process.

The exponential increase in the energy absorption for functionally graded samples differs from the linear increase of the energy absorption values observed for uniformly graded samples as reported by Sun et al. [4]. This change is due to the functionally graded nature of the samples. The functionally graded samples thus provide a more predictable energy absorption profile leading to more applications where undesirable failure modes in lattices structures can be eliminated and the benefit of the high specific energy absorption can be retained.

## 4. Conclusions

In this work, different functionally graded 316L steel lattice structures were designed and fabricated using LPBF process. The fabricated samples were then sandblasted to improve the surface finish of the samples. Surface morphology, quasi-static compression, and energy absorption capabilities were studied. The key findings of this study are:

- Sandblasting was found to be a viable post processing technique to remove the adhered powder particles and thus improve the surface finish.
- Quasi-static compression analysis reveals that the all TPMS samples fail layer-by-layer deformation. The samples expanded laterally by forming a radius along the edges.
- The Fisher–Koch S sample showed the highest yield strength and Young’s modulus. The Gyroid and the Primitive structures resulted in the lowest value for yield strength and Young’s modulus.
- The Gibson–Ashby model was determined for all the functionally graded TPMS structures based on the experimental results.
- The Neovius structure was found to absorb the most energy (479.62 MJ/m^3^) compared to the other TPMS structures up to 50% strain values.

The compressive and energy absorption behaviors will be beneficial for engineers to design and fabricate functionally graded 316L steel TPMS structures. The results indicate that the underexplored functionally graded Neovius and Fisher–Koch S offer greater energy absorption and yield strength values for impact and light weighting applications.

## Figures and Tables

**Table 1:** The chemical composition of 316L steel powder obtained from SLM solutions.

Element	Fe	Cr	Ni	Mo	Nb + Ta	Mn	Si	P	S	C	N	O
Mass fraction (%)	Bal	16–18	10–14	2–3	-	2	1	0.045	0.03	0.03	0.1	-

**Table 2:** The part densities of the sandblasted TPMS samples.

Sample	Diamond	Gyroid	Neovius	Primitive	Fisher–Koch S
Part Density (%)	98.87 ± 0.12	99.71 ± 0.23	98.82 ± 0.13	98.99 ± 0.13	98.03 ± 0.12

**Table 3:** Gibson–Ashby coefficient values for different TPMS lattices.

Coefficients	Given Range of Gibson–Ashby Coefficients	Diamond	Gyroid	Primitive	Neovius	Fisher–Koch S
C_1_	0.1–4	0.0396	0.0567	0.0285	0.0753	0.0717
C_2_	0.1–1	0.7369	0.6830	0.9053	0.7210	1.0554

**Table 4:** Energy absorbed values for different TPMS lattices.

	Diamond	Gyroid	Primitive	Neovius	Fisher–Koch S
Energy Absorbed (MJ/m^3^)	242.90 ± 6.1	296.67 ± 3.9	384.95 ± 7.1	479.62 ± 9.8	373.08 ± 6.4
